# Supplementary material for: The High Frequency of a G-Allele Variant of the FOXP3 Gene in Old Asian Cattle Breeds, Water Buffaloes, and Holstein Friesian Cows: A Potential Link to Infertility
Source: Animals (Basel). 2025 Aug 16;15(16):2407. doi: 10.3390/ani15162407 (PMC12382922; doi:10.3390/ani15162407)
Supplement: Supplementary file 1 [file animals-15-02407-s001.zip › animals-3777080-supplementary.pdf]

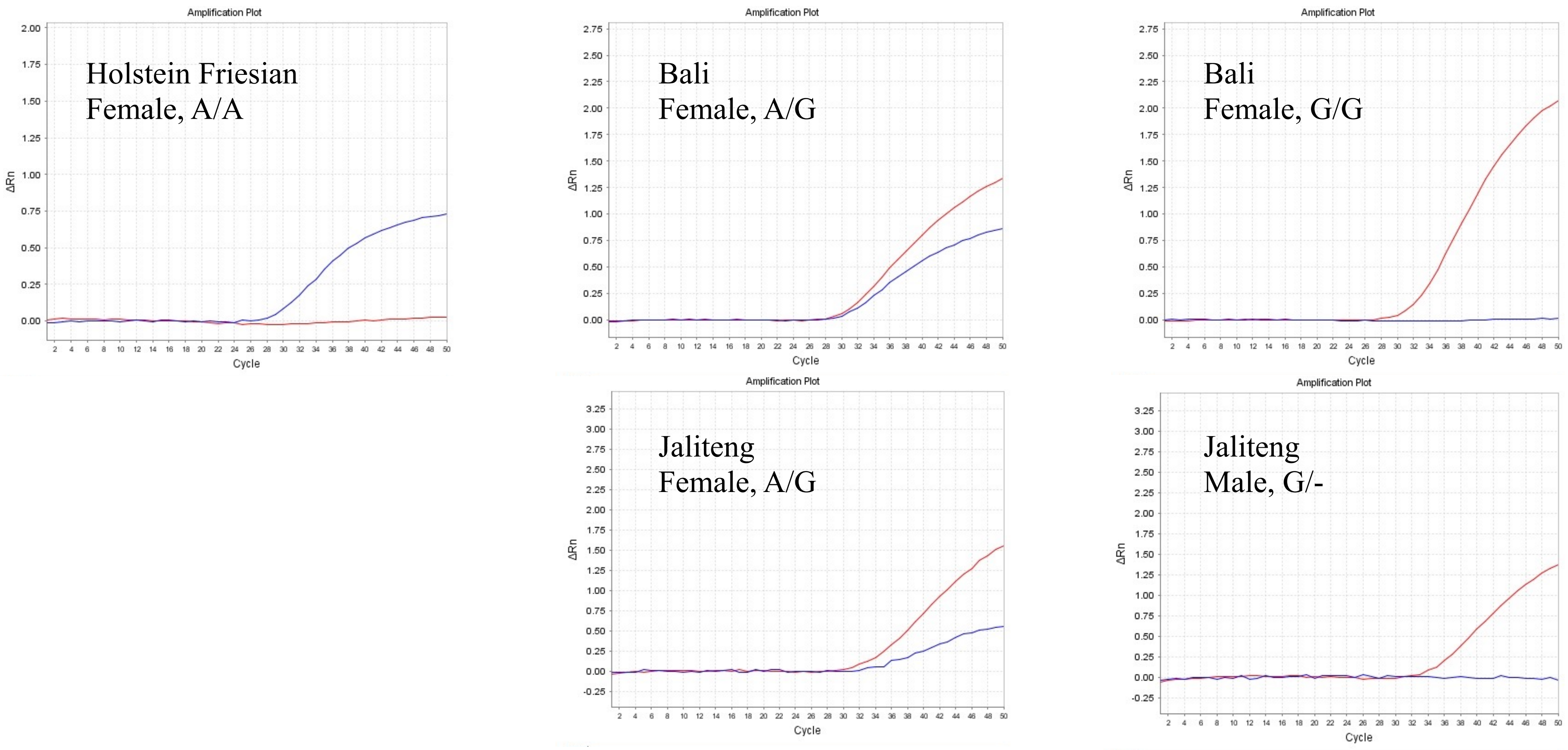

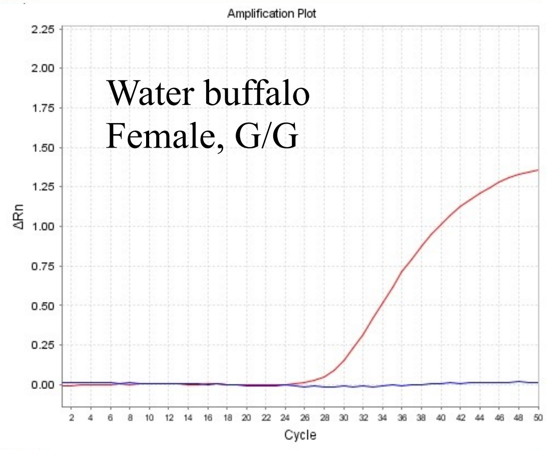

Figure S1. Representative real-time PCR amplification plots of the A/A, A/G, and G/G or G/- genotypes associated with an X-linked single nucleotide variant (NC_037357.1: g.87298881A>G, rs135720414) in the upstream of the *FOXP3* gene. Amplification is graphed as fluorescence intensity (ΔRn values) versus cycle number. ΔRn values denote the reporter dye signal, normalized to the internal reference dye, and adjusted for the baseline signal determined in the initial cycles of PCR. The blue and red lines signify amplification in the presence of A- and G-alleles, respectively.
